# Smoke-Free Public Policies and Voluntary Policies in Personal Settings in Tbilisi, Georgia: A Qualitative Study

**DOI:** 10.3390/ijerph13020156

**Published:** 2016-01-25

**Authors:** Carla J. Berg, Samantha A. Smith, Ta Misha Bascombe, Nino Maglakelidze, Lela Starua, Marina Topuridze

**Affiliations:** 1 Department of Behavioral Sciences and Health Education, Emory University School of Public Health, 1518 Clifton Road, NE, Room 524, Atlanta, GA 30322, USA; 2 Health Promotion and Wellness Directorate Army Public Health Center, 8506 Dawnridge Dr., Houston, TX 77071, USA; s.smith012@gmail.com; 3 Centers for Disease Control and Prevention, 1600 Clifton Rd, Atlanta, GA 30322, USA; tsbascombe@gmail.com; 4 National Center for Disease Control, 9 M. Asatiani st. Tbilisi, GA 0177, USA; nmaglakelidze@yahoo.com (N.M.); lela.sturua@ncdc.ge (L.S.); topuridzemarina@gmail.com (M.T.)

**Keywords:** tobacco control, secondhand smoke exposure, health policy, health disparities

## Abstract

Georgia has limited tobacco control policies, particularly in the area of smoke-free public policies, which may influence the adoption of smoke-free home rules. We qualitatively examined knowledge about and reactions to public and personal smoke-free policies among Tbilisi residents. In Spring 2014, we conducted six focus groups among 47 total participants—two among male smokers, one among male nonsmokers, two among female smokers, and one among female nonsmokers. Our sample was 48.9% male and 70.2% past 30-day smokers. Most believed that SHS was dangerous, with particular concern regarding the impact of SHS on children and pregnant women. Many had misconceptions about how to protect others from SHS and the effectiveness of some approaches. Many indicated that they had some type of home rules, but few reported a complete ban on smoking in the home. Even when some restrictions were in place, they rarely were effective or enforced. Common concerns about the partial smoke-free public policy in Georgia included its economic impact, perceived discrimination among smokers, and the policy being against the Georgian culture. These concerns were heightened when participants were asked about the possible implementation of a complete smoke-free policy. Educational programs are needed to promote smoke-free policies in Georgia.

## 1. Introduction

Cigarette smoking is a major global public health issue, with roughly 31.1% of men and 6.2% of women being daily smokers globally [1]. In fact, cigarette smoking is the second leading risk factor for death worldwide [2]. More than six million people die every year as a consequence of tobacco smoking [3]. Low- and middle-income counties (LMICs) are disproportionately affected by tobacco-related diseases and deaths. Roughly 80% of current smokers live in LMICs [3]. It is estimated that mortality will increase to 8.3 million a year by 2030, with 80% of these deaths occurring in LMICs [3].

One high-risk region for tobacco use is the area of the former Soviet Union [4]. A study of former Soviet Union countries (e.g., Armenia, Azerbaijan, Belarus, Georgia, Kazakhstan, Kyrgyzstan, Moldova, Russia, and Ukraine) documented that roughly 80% of men had a history of smoking and that there are drastically different smoking rates among men and women in these regions, with men having a much higher prevalence of smoking [4]. Georgia is one former Soviet Union country and a lower middle-income country [5] with high tobacco use prevalence. The 2010 Georgia STEPS report indicated that 53.0% of men and 4.4% of women were current smokers [6]. Moreover, more than 8500 of Georgia’s people are killed by tobacco-caused diseases annually [7].

Moreover, secondhand smoke exposure (SHS) is a major global public health problem. A recent study [8] found that, worldwide, 40% of children, 35% of female nonsmokers, and 33% of male nonsmokers were exposed to SHS in 2004. Moreover, they estimated that SHS exposure caused 603,000 deaths (about 1.0% of worldwide mortality), with nearly half of those deaths in women and over a quarter of them in children. Unfortunately, LMICs represent a high-risk group in terms of SHS exposure and the related morbidity and mortality [8].

Global health disparities related to tobacco use and SHS exposure are largely due to differences in tobacco control policy implementation, particularly in LMICs [9]. The World Health Organization (WHO) Framework Convention on Tobacco Control (FCTC) mandates that nations that ratify the FCTC implement a range of tobacco control policies. Public smoke-free legislation is a major evidence-based tobacco control effort promoted by the FCTC. Comprehensive smoke-free indoor air laws ban smoking of tobacco products in all indoor areas in worksites, restaurants, bars, and hotels and do not allow for separately ventilated areas. Public smoke-free policies are effective in reducing exposure to SHS, the initiation of tobacco use among young people, tobacco use prevalence, tobacco-related morbidity and mortality, and healthcare costs [10]. Among smokers, such policies are also associated with increasing cessation and cessation attempts and reducing cigarette consumption [10]. Unfortunately, only 18% of the world's population is protected by comprehensive national smoke-free laws [11].

Despite the ratification of the FCTC in December 2005, Georgia lags in terms of its adoption of all tobacco control policies mandated by the FCTC, including smoke-free public policies. Currently, there is no comprehensive policy in place and exceptions are made in almost every setting with the exclusions of healthcare facilities and universities. Even in settings where smoking is restricted or banned, the level of enforcement of these rules has not been documented. In efforts to progress in their level of smoke-free policies, in 2008, Georgia implemented a partial smoke-free policy applied to restaurants and bars.

Outside the context of smoke-free policies in public places, the home is a significant source of SHS exposure [12]. Smoke-free policies in personal places such as homes and cars are associated with reduced smoking among adults and a reduction of SHS among children and nonsmoking adults sharing those personal spaces [12,13]. Additionally, having smoke-free home policies is associated with reduced cigarette consumption [14], increased quit attempts, and reduced chance of relapse [14,15,16]. Therefore, adopting smoke-free policies in personal spaces can both protect individuals living in the home from SHS and help smokers quit. Disparities exist in terms of what population subgroups are less likely to have smoke-free home policies. For example, consistent predictors of household smoking bans include the presence of children, the presence of a nonsmoking adult in the home, and friends and family members who smoke [17,18], thus highlighting the social context of implementing such policies. The implementation and enforcement of smoke-free homes may indicate that a person is more likely to support smoke-free public places. This might be particularly true in areas that are lagging in public policy implementation, such as LMICs. Moreover, the rate of adoption of personal smoke-free policies may be impacted by the implementation of public smoke-free policies.

Given the aforementioned literature, we conducted a qualitative focus group study using Grounded Theory [19] to examine individual beliefs about SHS exposure, the process of adopting smoke-free policies in personal settings, and reactions to the partial public smoke-free policy that was implemented in Georgia in 2008 among Georgian adult smokers and nonsmokers.

## 2. Experimental Section

### 2.1. Procedures and Participants

This study was approved by the Institutional Review Boards of Emory University and the National Centers for Disease Control and Public Health in Georgia. In Spring 2014, we conducted six focus groups among a total of 47 participants—two among male smokers, one among male nonsmokers, two among female smokers, and one among female nonsmokers in Tbilisi.

Focus groups are well-suited to explore individual subjective experiences and attitudes, particularly related to concepts or phenomenon that have not been well explored previously [20,21,22,23]. This is relevant given the lack of prior research related to perceptions of public smoke-free policies in Georgia and the process of adopting smoke-free homes in this context. Moreover, focus groups are more effective than in-depth interviews in simulating real-world responses to questions, getting an overview of attitudes, exploring consensus or lack thereof, comparing and contrasting groups (e.g., genders, by smoking status), and reducing social desirability in response to the interviewer [20,21,22,23]. Thus, this qualitative approach was selected. In the context of our qualitative research, we leveraged Grounded Theory [19], which is a systematic methodology involving the construction of theory through the analysis of data. This approach typically begins with a question, or even just with the collection of qualitative data and then relies on the analyses to synthesize the findings.

Participants were recruited through fliers and posters posted throughout Tbilisi, the capitol city of Georgia. Interested individuals called the research staff and were confirmed for eligibility. Eligibility criteria included: (1) between the ages of 18 and 65 years old; and (2) spoke Georgian. If individuals were deemed eligible, they were asked if they currently smoke tobacco on a daily basis, less than daily, or not at all. Those that reported daily smoking or less than daily smoking were categorized as current smokers [24]. Participants were scheduled for a focus group session based on their gender and smoking status. Once saturation was reached, recruiting participants was discontinued as recommended by Glaser and Strauss [25]. The range of sample size for the focus groups was 7 to 9, with focus group size being a median of 8.5 participants.

Focus groups were conducted in conference rooms at the National Centers for Disease Control and Public Health in Tbilisi. Prior to the focus group discussion, participants completed the informed statement of consent and a brief survey. Each participant was compensated with a top-up mobile balance worth 10 Georgian Lari for participation. The focus groups were designed based on methods proposed by Morgan and Krueger [26]. Two moderators (MT and a Masters of Public Health level staff) experienced in qualitative methodology and familiar with the topic of tobacco control guided the groups through a semi-structured focus group discussion that lasted approximately 90 min. All sessions were audio-taped, observed by a research assistant who recorded field notes, and transcribed by a professional service. After each session, the moderator and research assistant debriefed. No changes were made to the interview guides as a result of debriefing. The interviewer recorded field notes to aid in data analysis. After the initial six focus groups planned were completed, the research team determined that saturation was reached, and recruitment was discontinued.

### 2.2. Instrumentation

*Semi-structured Interview Guide*. The semi-structured focus group interview guide was developed by the research team and was pilot tested through mock interviews among research staff members. It explored perceptions of SHS, experiences with and reactions to public smoke-free policies, and personal practices regarding smoking in homes and cars. Example questions regarding SHS included: “How harmful do you think SHS is? Do you believe that SHS can cause health problems for nonsmokers? For children? What kind of health problems?” Questions regarding restrictions in personal settings included: “What rules do you have in your home and car about smoking cigarettes? How have public restrictions impacted the rules in your home and car? How did you decide to implement restrictions in your home or car? How strictly do you adhere to these rules? How do others respond to your rules?” Questions regarding experiences with public smoke-free policies included: “What do you know about the policy in Georgia regarding smoke-free areas in restaurants and bars that went to effect in 2008? What do you think of the policy that went into effect? What reasons might you have for opposing the policy? What reasons might you have for supporting the policy? How effective do you think no-smoking areas in protecting people from the harms of SHS? To what extent do you think this policy has actually been enforced?” 

*Quantitative Assessment*. A short questionnaire was used to assess sociodemographics and tobacco use behaviors. Participants were asked to report their age, gender, number of years of education, their monthly income in Lari, their employment status (employed part-time, employed full-time, unemployed, student, homemaker, retired, unable to work, or disabled, other), their relationship status (married, living with a partner, single/never married, divorced, or widowed), and the number of people in the home [27].

In terms of tobacco use characteristics, participants were asked if they used tobacco in their lifetime and if they currently smoke tobacco on a daily basis, less than daily, or not at all. Those that reported daily smoking or less than daily smoking were categorized as current smokers [24]. Current smokers were also asked to report the age at which they had their first cigarette, the age at which they began smoking regularly, the number of days in the past 30 days that they smoked, and the number of cigarettes they smoked per day (CPD) [24].

### 2.3. Data Analysis

Focus group interviews were transcribed verbatim by a professional transcription service, and text files were imported into qualitative analysis software. NVivo 10.0 (QSR International, Cambridge, UK; Boston, MA, USA) was used for text coding and to facilitate the organization, retrieval, and systematic comparison of data. The research team was guided by the COREQ checklist when conducting and analyzing interview data [28]. Transcripts were independently reviewed by two research project staff members, both of whom were Master of Public Health level staff trained in qualitative analyses. These two coders generated preliminary codes using an inductive process. The research team used an iterative process to develop a master coding structure [29,30]. Primary (*i.e.*, major topics explored) and secondary codes (*i.e.*, recurrent themes within these topics) were then clearly defined in a codebook that was used to independently code each transcript. Coders resolved any discrepancies through discussions. Intra-class correlations for context were 0.92. Themes were then identified and agreed upon between the PI and the coders, and representative quotes were selected and identified by group (FG1-2 = female smokers; FG3-4 = male smokers; FG5 = female nonsmokers; FG6 = male nonsmokers).

Descriptive statistics from the short questionnaire were computed using means and standard deviations for continuous variables and frequency and percentage for categorical variables. Bivariate analyses were computed using t-tests for continuous variables and Chi-squared tests for categorical variables. Quantitative data were analyzed using SPSS 19.0 (IBM Corporation, Armonk, NY, USA).

## 3. Results

Table 1 provides an overview of our sample in terms of sociodemographics and tobacco use characteristics. Our sample was on average 25.60 years old (SD = 5.49), 48.9% male, 34.0% married or living with a partner, and 22.6% with children in the home. They had an average of 13.49 years of education (SD = 3.96), and 34.0% were employed. Current (past 30 day) tobacco users comprised 70.2% of the sample. With the exception of current smokers having fewer people in the home (*p* = 0.032), there were no differences in sociodemographics between the smoker and nonsmoker participants.

**Table 1:** Focus Group Participant Characteristics and Bivariate Analyses of Nonsmokers *vs*. Smokers, *N* = 47.

Variable	All Participants	Nonsmokers	Smokers	*p*-Value
	M (SD) or N (%)	M (SD) or N (%)	M (SD) or N (%)	
Sociodemographic Factors				
Age (SD)	25.60 (5.49)	23.64 (4.81)	26.42 (5.61)	0.113
Gender (%)				0.412
Male	23 (48.9)	6 (42.9)	17 (51.5)	
Female	24 (51.1)	8 (57.1)	16 (48.5)	
Number of years of education (SD)	13.49 (3.96)	13.64 (3.13)	13.42 (4.31)	0.865
Income per month (Lari) (SD)	1250. 00 (1340.30)	1157.13 (604.74)	1289.39 (1558.33)	0.761
Employment status (%)				0.237
Employed full- or part-time	16 (34.0)	3 (21.4)	13 (39.4)	
Unemployed	10 (21.3)	4 (28.6)	6 (18.2)	
Homemaker	13 (27.7)	6 (42.9)	7 (21.2)	
Other	8 (17.0)	1 (7.1)	7 (21.2)	
Relationship status (%)				0.199
Married/living with partner	16 (34.0)	3 (21.4)	13 (39.4)	
Other	31 (66.0)	11 (78.6)	20 (60.6)	
Number of people in the home (SD)	3.87 (1.19)	4.46 (1.27)	3.64 (1.08)	0.032
Tobacco Use				
Age of first cigarette (SD)	-	-	15.42 (7.25)	-
Age began smoking regularly (SD)			17.83 (3.36)	-
Days smoked, past 30 days (SD)	-	-	24.91 (9.96)	-
CPD (SD)	-	-	13.33 (10.38)	-

### 3.1. Secondhand Smoke

Most participants believed that SHS was dangerous. There was particular concern regarding the impact of SHS on children and pregnant women. For example, one female smoker (FG1) said, “I have heard and read that it’s far more dangerous for children than for smokers themselves”. Along the same lines, one male smoker said, “One should not smoke at home, in the presence of a pregnant woman and of a child”.

Interestingly, a couple of participants felt that protecting children from SHS was impractical. For instance, one female smoker (FG1) said, “Honestly, I believe that it’s ok to smoke in front of children, as they have to get used to it, as smoking is an everyday activity”.

There were misconceptions about how to protect others from SHS and the effectiveness of some approaches to do so. For example, one female smoker (FG2) said, “I ask a non-smoker if the smoke bothers him/her. If he/she tells me that it bothers, for example if we are in the car, I will either roll down the window or act in a way that smoke does not get in his/her face”. These misconceptions were reflected in discussions regarding smoke-free policies or rules regarding smoking in private residences and personal vehicles.

### 3.2. Smoke-Free Policies in Personal Settings

*Lack of Home Policies or Exceptions to Policies.* In terms of voluntary policies in homes, many indicated that they had some type of rules, but few reported a complete ban on smoking in the home. Among those that said that “smoking wasn’t allowed in their home”, they frequently commented on places where smoking was allowed in the home, with common places including kitchens or bathrooms. One female smoker (FG2) said, “It is forbidden to smoke in my home, so I go to the toilet and smoke”.

Another female smoker (FG1) indicated, “At my place, no one is allowed to smoke. I’m a smoker and so is my husband, but for smoking, you either go to the balcony or use the kitchen”.

While many participants reported that they restricted smoking particularly when children were present, they commonly reported that they made exceptions when they had guests in their home. One female smoker (FG2) said, “People don’t smoke at my place, but whenever a guest is visiting, I don’t prohibit smoking. We respect the guests, and I do not prohibit them to smoke. I know that it is bad, but I can’t prohibit it”.

In addition, even when some sort of restrictions were in place for guests, they rarely were effective. Reflecting an ineffective attempt to reduce SHS exposure, one female smoker (FG2) said, “When a guest is visiting I make him/her sit at the window; he/she will be disturbed but he/she won’t smoke for the second time”.

Highlighting the difficulties of enforcing restrictions when guests visit, one female nonsmoker (FG5) said, “Some people ask if they should go out on a balcony, and at the same time they are already hold a lit up cigarette. At that point, you can’t chase them outside”.

*Impact of Others’ Rules Regarding Smoking in the Home.* In terms of reactions to others’ rules about smoking in their home, several participants who were smokers indicated that they avoided going to those places. For example, one female smoker (FG2) said, “Yes, it bothers me (to go where smoking is not allowed). I never go to those places where I cannot smoke. There are some families where no one smokes, and I don’t like to go to their homes. In my family, we smoke, and our guests can smoke as well, but not in the room where our child sleeps”.

Another female smoker (FG2) said, “I regularly find myself in those kind of situations (where I cannot smoke), for example at my friend’s place, or at my relative’s place, or at my mother in law’s place. And since I can’t smoke in those places, I don’t go there that often. I always argue regarding this with the taxi drivers, if he/she tells me that he/she does not smoke and asks me not to smoke as well”. This participant and several other smokers indicated that they resisted requests not to smoke in both personal and public settings.

Participants also indicated that going to homes where smoking commonly takes place increased the likelihood that they would smoke as well. For example, one female smoker (FG2) said, “I have a friend. Both mother and daughter smoke. And at their place there is an atmosphere in which I somehow have to smoke. There is no other way there. I don’t feel comfortable there otherwise”.

### 3.3. Public Smoke-Free Policies

*Concerns about and Opposition to Public Smoke-free Policies*. Themes that emerged regarding concerns about partial smoke-free public policy reported by participants included the economic impact of the policy, the discriminatory nature against smokers of such policies, and the policy being against the culture of Georgians. These concerns were heightened when participants were asked about the possible implementation of a complete smoke-free policy.

Reflecting one concern highlighted by several participants regarding the impact of smoke-free policies on the economy, one female smoker (FG1) stated, “A 100% ban will damage restaurant owners in terms of economy and the government, too, as owners bring money. If you don’t want to lose clients it should be 50%–50% among smoking and nonsmoking areas. This percentage shows how probable it is to ban smoking in Georgia”.

Weighing the costs *versus* benefits of smoke-free policies, one female smoker (FG1) said, “If smoking is banned, it’ll damage the economy but the health condition will improve”.

Many participants also indicated that having expansive smoke-free policies in Georgia is impossible due to the culture and smoking prevalence of the country. One female smoker (FG1) said, “The country’s psychology should be considered. The rate of smoker women in Georgia is one of the highest, at the same time Georgia is one of the most conservative countries”.

One female smoker (FG1) said, “I like a lot the idea of dividing cafes and bars into smoking and nonsmoking areas, but in Georgia, I’m afraid, it’s impossible in Georgia”.

Reflecting the same concern, one female smoker (FG1) said, “I think it’s unrealistic to think about smoking areas outside in Georgia due to the mentality that is here. Nobody will go out to smoke during winter, put on coats and then take them off again and return to a bar or a restaurant”.

Another theme that emerged was feeling as though smoke-free policies discriminated against smokers, with a few participants indicating such a concern. One female smoker (FG1) stated, “Well, we can go out and defend our rights like LGBT. In a way, it’s discriminatory, and I don’t like it”.

*Support for Public Smoke-free Policies*. Despite these concerns, many participants—both nonsmokers and smokers—were in favor of the partial smoke-free policy. They highlighted the importance of protecting people from SHS, the pleasure of being in public without being exposed, the impact such policies will have on population health, and the impact it could have on social norms regarding smoking. One female nonsmoker (FG5) said, “There are some places where there are designated areas for nonsmokers, and this is very pleasant. One gets the feeling that, besides smokers, the owners also start caring about nonsmokers…. At the same time this will be a good impetus for smokers to smoke less and maybe stop smoking all at once”.

Another female smoker (FG2) said, “Certain organizations have limitations, and I like it, since this helps to develop a certain culture and teaches one to abide the laws. There are separate places in the cafes as well, where smokers and non-smokers sit separately”.

One female nonsmoker (FG5) said, “(A public smoke-free policy) will work for sure. Many people will feel lazy to go out, and they won’t smoke”.

There were several smokers who indicated that they liked the impact it made on their smoking. One female smoker (FG2) said, “If one cannot smoke, I won’t smoke either. I ask where it is possible to smoke. If it is impossible to smoke, I will go outside or not smoke at all”.

*Ineffective Enforcement and Measures to Reduce SHS Exposure.* A major theme that emerged was that measures being used in the context of the partial ban were not effective in protecting nonsmokers from SHS. One male smoker (FG4) said, “I remember a new restaurant opened, and when I went there. I was asked if I preferred smokers’ or nonsmokers’ part. When I looked, it was one same hall divided into two parts. I once sat with nonsmokers, but the smell came to our area, too”.

A different male smoker (FG3) reported, “When I was in the mall, there was a place for nonsmokers as well as the place for smokers. I was sitting there, a smoker, and beside me there was a non-smoker, so this is not following the rules. We should be separated properly. When I enter a place, I should have a feeling of controlling myself regarding where I smoke”.

Another major theme that emerged was lack of enforcement. Most participants reported having violated smoke-free policies at work, in public transportation, or other public settings, or witnessing the rules being broken. For example, one male nonsmoker (FG6) said, “There are restaurants who have separate places for smokers and nonsmokers; only in some places is this law respected”.

Participants were forthcoming in telling about situations in which they would violate smoke-free policies. For example, one female nonsmoker (FG2) said, “When I was working, there was a bar under the office where I would go to, and we girls would drink coffee there and smoke. But when we would not go there, we still managed to smoke at work, and we were taking a risk because smoking could have been the reason for us to lose our jobs”.

Further compounding the issue of enforcement, many participants indicated that they would not say anything in situations where they were being exposed to SHS, even in the context of a nonsmoking area or vehicle (e.g., the minibuses). One female smoker (FG2) said, “Whenever I sit in a café, in a non-smoking area, and I feel that someone has smoked near me, I won’t take offense personally. And I won’t offend anyone because I think that this kind of person is ill, who needs a cigarette and can’t do without it”.

A female nonsmoker (FG5) said, “When you are in a taxi you can tolerate for 5–10 min, but when you travel on a long distance and the Marshutka (minibus) driver smokes, as well as people sitting next to him and the smoke gets into the back cabin, and plus it is winter and everything is closed, then this becomes very hard. And I become very angry. But nobody pays attention and everyone is silent. But in reality many have the feeling of protest”.

## 4. Discussion

Overall, current findings indicate that cigarette smoking and SHS exposure are critical public health problems in Georgia. One major finding was a lack of empowerment to protect oneself from SHS. A recent national survey on Georgian adults [31] indicated that, despite the fact that over 93% of participants believed SHS exposure is harmful, over four-tenths were exposed daily, with an additional 12% being exposed several times a week. Unfortunately, there were several reports of both smokers and nonsmokers avoiding conflict or potentially offending smokers in regard to being exposed to SHS in public settings or enforcing their personal home smoking rules.

Moreover, prior research has found that four-tenths of Georgian adults are exposed to SHS in their home daily, and in fact, only 14.3% indicated that smoking was never allowed in their home [31]. It is likely that even those that reported complete policies still face SHS exposure in the home, given the number of people in the current qualitative study that reported that they never allowed smoking in the home but then articulated several exceptions in terms of circumstances and specific locations within the home. It is also interesting to note that, perhaps related to the fact that few homes prohibit smoking in the home, participants in this study indicated that they would avoid going to homes where smoking was prohibited or might be more likely to smoke in homes where smoking was allowed.

This prior study [31] also documented that public settings were a significant source of SHS exposure, with a quarter to a third of participants reporting at least a day a week of exposure to SHS in the workplace and in public settings. This is not surprising given the weak public smoke-free policies in place in Georgia. However, this study also documented public sentiment in support of greater public smoke-free policy implementation, with the majority of both nonsmokers and smokers supporting smoke-free public policies across most settings, with the greatest opposition being to such policies in restaurants, bars, and patios [31]. Bars and restaurants are two public settings that have proven to be a challenge for passing smoke-free legislation [32]. In Georgia, only partial smoke-free policies apply to these settings such that a nonsmoking section is required in these establishments.

Some of the common concerns about the partial smoke-free policy included concerns about the economic impact of the policy, the perceived discriminatory nature of the policy, and the difficulties in consolidating such a policy with the culture of Georgia, which includes a high smoking prevalence. In relation to the former, evidence suggests that smoke-free policies do not have an adverse economic impact on businesses and may have a positive impact in some contexts [10]. However, more research is needed in LMICs with high smoking prevalence to ensure these data are relevant.

One of the major overarching findings in the current study involved measures being taken to reduce harm, both in private and in public settings, that are ineffective. In relation to the home, participants frequently reported limiting smoking to certain rooms or using windows or fans to circulate smoky air in order to reduce the impact of SHS exposure. While the public health goal is complete smoke-free home policies, partial bans have been found to decrease SHS exposure [33,34]. As such, it is important to acknowledge and promote some harm reduction measures. In terms of partial smoke-free policies, participants reported multiple experiences of the partial policy being ineffectively implemented, such that SHS was clearly not prevented within nonsmoking areas. Note that the 2006 U.S. Surgeon General’s report concluded that strategies such as opening windows, air ventilation, and separating smokers from nonsmokers do not afford complete protection and that only preventing smoking indoors can fully protect nonsmokers from effects of SHS [12]. As such, better education and regulation regarding appropriate measures for reducing SHS exposure in Georgia are needed.

Moreover, the lack of enforcement of policies that do exist also suggests high levels of SHS exposure in spite of any policies that are implemented. Given the limited smoke-free policies in place in Georgia and the difficulties enforcing those in place, there are two major concerns. First, the level of SHS exposure and related morbidity and mortality is an obvious concern. Second, the challenges facing the culture of Georgia in terms of shifting social norms regarding smoking are surmounted by these policies being unenforced.

Current findings have important implications for research and practice. First, research must change the way in which they assess smoke-free home policies. It is clear that asking individuals if they allow smoking in the home is limited, as people may indicate that they do but have multiple exceptions, particularly in contexts like Georgia where cultural norms are conducive to smoking. In addition, future research should examine the processes that impede the voluntary adoption of such policies in personal settings and adoption of comprehensive public smoke-free policies in Georgia. For public health practitioners, our findings suggest that Georgians are largely in support of public smoke-free policies and the inefficiency of measures taken in the context of partial smoke-free policies. Thus, this empirical evidence should be leveraged toward garnering political will to implement such policies, particularly within the context of the significant public health costs of SHS-related morbidity and mortality in Georgia. Moreover, practitioners must educate the general public about the limitations of certain harm reduction measures in protecting nonsmokers and children from the harms of SHS. Collectively, these findings provide a foundation to inform the activities of public health practitioners to further the agenda of public smoke-free policy adoption.

## 5. Limitations

This study has some limitations. First, while participants were recruited for focus groups to represent each gender and both smokers and nonsmokers, findings from this small sample may not generalize to other Georgian adults. In particular, it is important to note that our sample was younger than the general Georgian adult population and had a higher rate of unemployment (*vs*. 14.6% in Georgia in 2013 when recruitment began [35]). Also, the self-reported assessments limit the extent to which we can account for bias. Finally, we did informally monitor reasons for nonparticipation and found that scheduling was the most common reason, which is common in focus group recruitment. As such, our results must be interpreted with caution. Despite these limitations, these findings are important given the dearth of published research on correlates of receptivity to public smoke-free policies and implementation of voluntary smoke-free policies in individual homes and cars among Georgian adults.

## 6. Conclusions

In Georgia, efforts must promote smoke-free policies, particularly among male smokers, in order to reduce SHS exposure, smoking rates, and ultimately the related morbidities and mortality. Georgians are experiencing high levels of exposure to SHS both in public and private settings. While they are in support of adopting more rigorous public smoke-free policies, legislation has yet to adopt such policy. Moreover, the limited voluntary implementation of smoke-free policies in homes and cars warrants intervention efforts.
